# An Unusual Presentation of Acute Cholecystitis due to a Gallbladder Volvulus in a Young Female: A Case Report and Review of the Literature

**DOI:** 10.7759/cureus.21275

**Published:** 2022-01-15

**Authors:** Nattawut Keeratibharat, Jirapa Chansangrat

**Affiliations:** 1 School of Surgery, Institute of Medicine, Suranaree University of Technology, Nakhon Ratchasima, THA; 2 School of Radiology, Institute of Medicine, Suranaree University of Technology, Nakhon Ratchasima, THA

**Keywords:** abdominal pain, acute abdomen, cholecystitis, gallbladder volvulus, gallbladder

## Abstract

The gallbladder volvulus is a rare entity of acute abdomen and a life-threatening condition that requires urgent surgery. The etiology of gallbladder volvulus is still unclear, and there is intraoperative evidence of a floating gallbladder that leads to a twisting of its pedicle, thus causing gallbladder ischemia and necrosis. Gallbladder volvulus is more frequently encounter in elderly females. We report a case of a 27-year-old female who presented with a clinical manifestation consistent with acute cholecystitis. Radiologic finding demonstrated displacement of the gallbladder body and fundus from the gallbladder fossa into the gastrohepatic recess. An emergency laparoscopic cholecystectomy was performed, and intraoperative findings revealed a gangrenous free-floating gallbladder that displaced to the dorsal sector of the left lobe liver. The critical view of safety was able to identify clearly and the gallbladder was removed safely with laparoscopic approach. While many previous cases have been reported, diagnosis of gallbladder volvulus remains difficult especially in young adults. Prompt diagnosis and surgery in cases of acute cholecystitis due to gallbladder volvulus are important to avoid gallbladder necrosis and perforation and result in a good outcome.

## Introduction

Gallbladder volvulus is a rare condition, with approximately 500 reported cases in the literature [1] since it was first described by Wendel in 1898 [2]. Twisting of the gallbladder around its pedicle causes an occlusion of the vascular and bile flow, leading to rapid gallbladder necrosis and perforation. The pathophysiology of acute calculous cholecystitis is the obstruction of the cystic duct with preserved normal arterial supply, which differs from the pathophysiology of gallbladder volvulus causing acute cholecystitis. Gallbladder volvulus is predominant in the elderly group, with a median age of 77 years, and is more frequent in females than in males (4:1) [3]. Physical examination to distinguish between acute cholecystitis and gallbladder volvulus is complicated by the preponderance of the latter condition in elderly patients. Preoperative diagnosis without imaging is difficult. Less than 10% of cases can be diagnosed before surgery [4]. Physical examination and radiological findings of acute calculous cholecystitis and gallbladder volvulus are similar. The treatment of choice for gallbladder volvulus is emergency detorsion and cholecystectomy, while acute cholecystitis usually does not require immediate surgery. A delay in treatment leads to an increased risk of sepsis secondary to gallbladder ischemia, necrosis, and perforation, with subsequent high mortality.

Gallbladder volvulus is very rare in young adults. In this report, we present a case of a 27-year-old female patient whose gallbladder torsion was diagnosed preoperatively and successfully treated with laparoscopic surgery in our center. We also review the existing literature on gallbladder volvulus and present recent summary of preoperative investigation, surgical techniques, and possible complications. Because of the increase in gallbladder volvulus incidence since 2000, we would like to contribute this case report and literature review to promote awareness of diagnosis and prompt surgical treatment of gallbladder volvulus.

## Case presentation

A 27-year-old healthy female presented to our emergency department with a two-day history of right upper quadrant (RUQ) pain. The pain was described as dull and intermittent pain localized to the RUQ and epigastric area. The pain was not associated with meals. She was afebrile, and her vital signs were within the normal limit. Abdominal examination revealed that the abdomen was soft, and there was no palpable mass, with localized mild tenderness at the epigastric area. Laboratory studies, including complete blood count, liver enzymes, and pancreatic enzymes, were all within the normal range. Abdominal ultrasonography was performed at the emergency department, showing a well-distended gallbladder without gallstone and cholecystitis features. An initial diagnosis of severe dyspepsia was made, and the patient was admitted to the hospital for symptomatic treatment and further workup. After around 12 hours of admission, the patient complained of increasing abdominal pain and fever.

**Figure 1:**
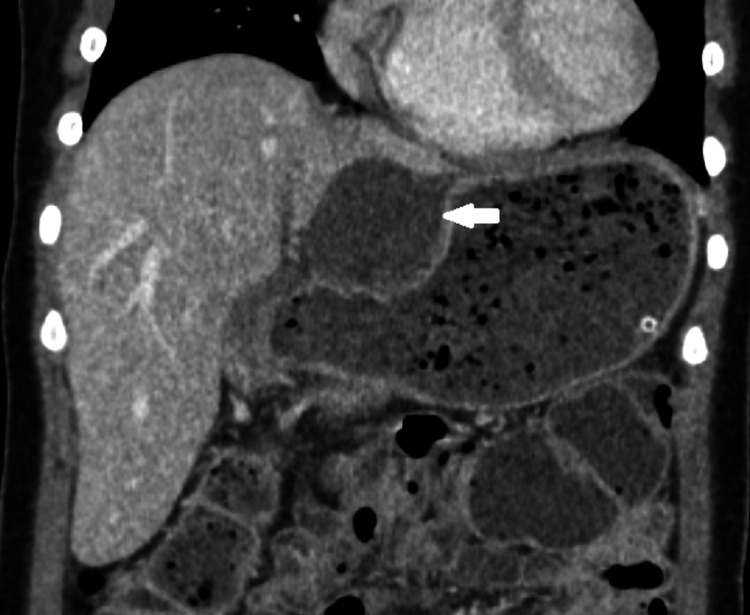
Coronal image showing displacement of the gallbladder into the hepatogastric recess (arrow).

**Figure 2:**
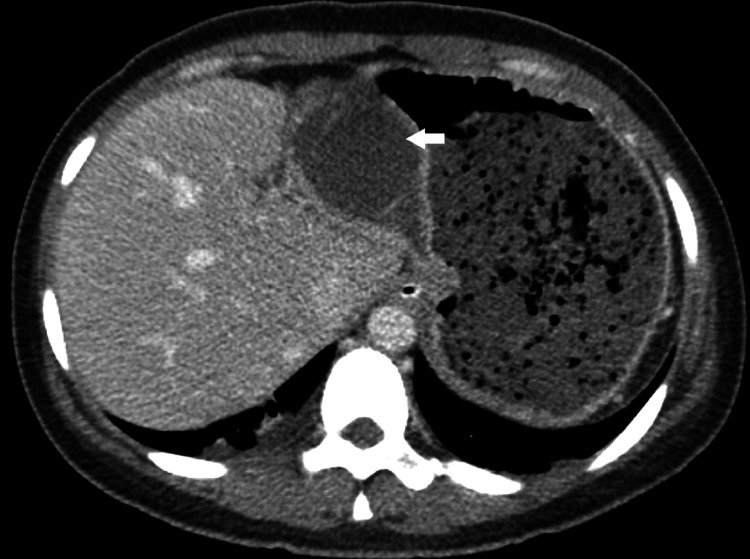
Axial image showing gallbladder in the hepatogastric recess, with diffuse wall thickening with diminished enhancement (arrow).

Abdominal examination revealed tenderness and guarding of RUQ. However, Murphy’s sign was negative. Laboratory tests were obtained, showing an elevation of leukocyte count of 13,300 /µL and neutrophil accounting for 86%. Subsequent abdominal computed tomography (CT) demonstrated displacement of the gallbladder body and fundus from the gallbladder fossa to gastrohepatic recess. There was marked distended gallbladder and diffuse wall thickening of the gallbladder without opaque stones. Diminished enhancement of the gallbladder wall was also noted (Figures 1, 2). Based on these findings, the patient was diagnosed with gallbladder volvulus and acute acalculous cholecystitis. Intravenous fluid resuscitation, antibiotics, and analgesics were administered. Then, the patient was scheduled for emergency laparoscopic cholecystectomy.

**Figure 3:**
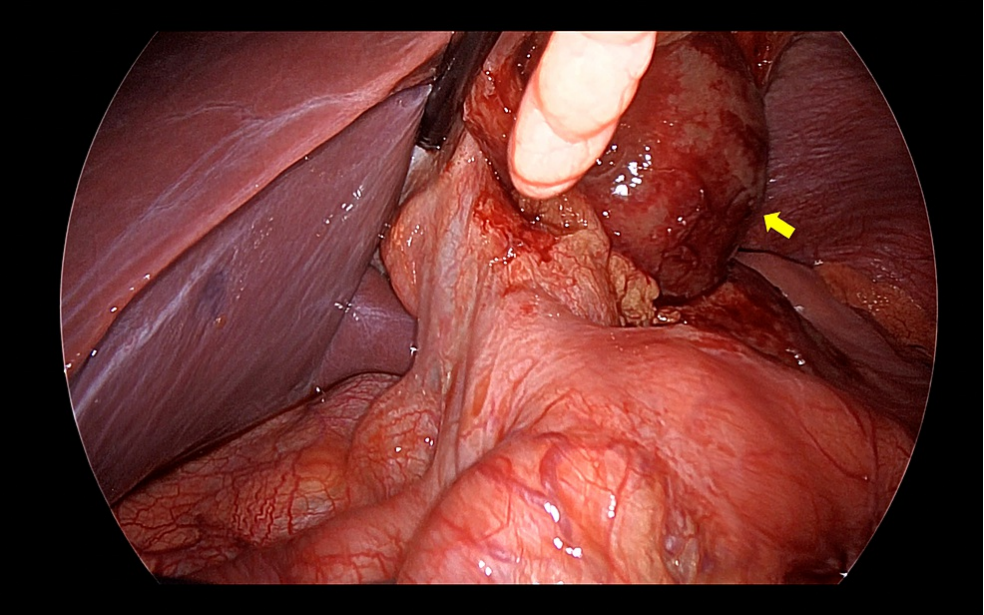
Intraoperative view of the gallbladder volvulus that displaced to the left side of the falciform ligament (arrow).

**Figure 4:**
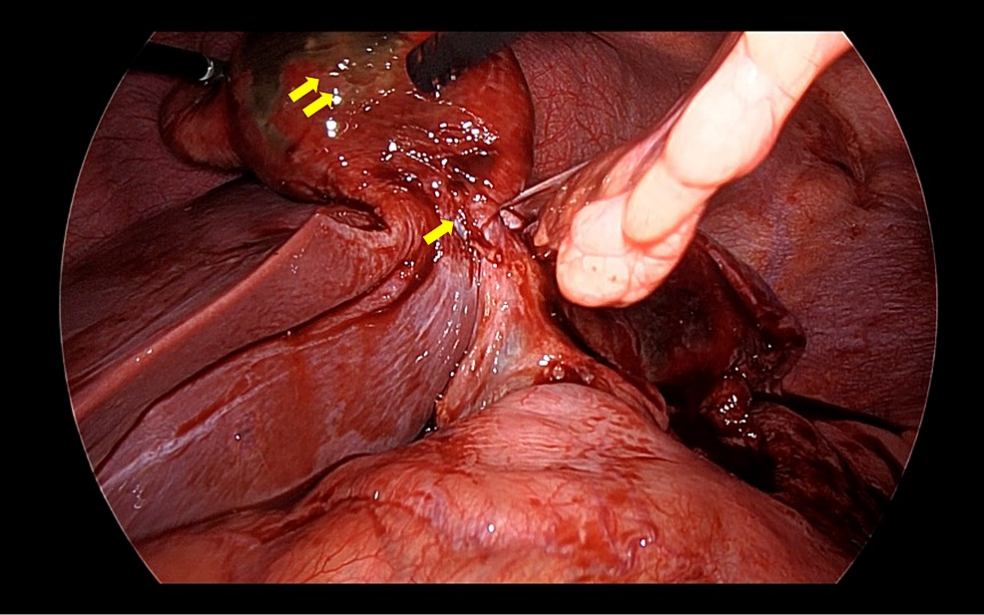
Intraoperative view of the gallbladder pedicle after isolation and detorsion (one arrow) and gangrenous part of the gallbladder (two arrows).

**Figure 5:**
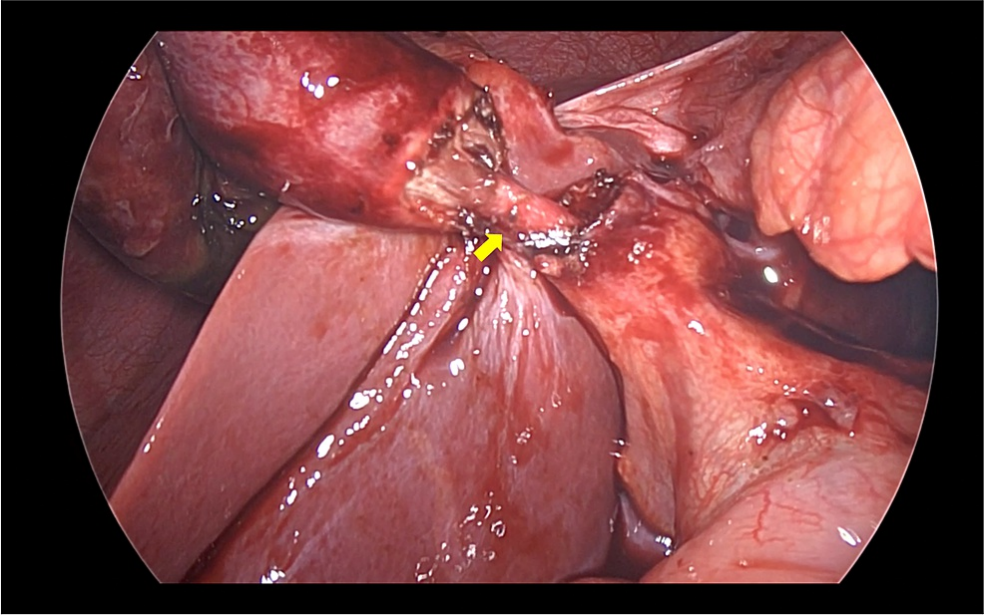
Intraoperative view of the cystic duct after dissection (arrow).

Operative findings revealed a distended gangrenous gallbladder that displaced to the gastrohepatic recess with stomach walled-off (Figure 3). After successful isolation and detorsion, the gallbladder was noted to be free floating and completely rotated anticlockwise (360°) around its pedicle (the cystic duct and artery) (Figure 4). Before division of the cystic duct and artery, the critical view of safety was established. Then, cholecystectomy was performed (Figure 5). The gallbladder was placed in a laparoscopic retrieving bag and removed from the intra-abdominal cavity via the umbilical port. The postoperative course was uneventful. The patient was discharged without complications. Postoperative liver enzymes were within the normal range. Histopathology was consistent with gangrenous cholecystitis without evidence of gallstone.

## Discussion

Pathophysiology and risk factors

To date, there is no known exact cause of gallbladder volvulus. Multiple conditions associated with an increased risk of gallbladder volvulus have been proposed. Anatomical variation of the gallbladder is the major risk factor. Normally, the gallbladder lies beneath the liver, immediately adjacent to the interlobar fissure. Anteriorly, the gallbladder is covered by an adventitia that is fused with the capsule of the liver. Posteriorly and on the tip, it is covered by the visceral peritoneum. These supportive structures do not allow the gallbladder to move freely. However, if a hallow caudal bud lags behind the movement of cranial bud during development, a floating gallbladder will occur. The floating gallbladder is covered with peritoneum and suspended from the liver surface by the mesentery. There are two types of floating gallbladder: type A, in which the mesentery supports the gallbladder and cystic duct, and type B, in which the mesentery supports the cystic duct only [5]. This anatomical variation leads to gallbladder mobility, thus increasing the risk of volvulus. The variant is reported to be around 1.3% [6]. Another strong predisposing factor is the loss of supporting structures, which are fat, fibrous connective tissue, and liver parenchyma, resulting in free hanging of the gallbladder [7,8]. Conditions associated with these factors are aging, excessive weight loss, and atrophy of the liver. Kyphosis is another contributing factor to gallbladder volvulus, as the condition displacing the gallbladder in a more dependent position [9]. The atherosclerosis of the cystic artery makes the artery hard. The endpoint of the hardened vessel would act as a fulcrum of torsion. Reported mechanical provocative factors are increased peristalsis, particularly of bowel loops adjacent to the gallbladder, sudden shift in body position, and blunt trauma. Hormonal inciting factor is increased cholecystokinin, which leads to gallbladder peristalsis and facilitates gallbladder torsion [10].

Gallstones show no significant correlation with gallbladder volvulus. Around 70%-80% of patients with gallbladder volvulus have no gallstone at the time of diagnosis [11]. In available reported data, patients’ ages ranged from 3 to 96 years, with two peaks of incidence: a minor group (16%) in pediatric patients (<18 years) and a predominant group (84%) in elderly patients (>70 years). The preponderant gender is male in the pediatric group and female in the elderly group, with a ratio of 5:1 [3,11,12]. The overall diagnosis of female patients is 76.2% due to it being predominant in the elderly age group, and the diagnosis of male patients is 23.8%. Congenital factors seem to be the causes for gallbladder volvulus in pediatric patients, consistent with the small number of the associated anatomical variation, whereas the physiological changes associated with aging appear to be responsible for gallbladder volvulus in adults. There was no significant relationship between the direction of torsion (clockwise or counterclockwise) and the degree of torsion (180°, incomplete torsion; >180°, complete torsion) [3].

Pathophysiology after the torsion

The range of volvulus can be divided into complete (>180°) volvulus and incomplete (<180°) volvulus. Incomplete volvulus may be misdiagnosed as biliary colic, whereas complete volvulus produces continuous, sudden RUQ pain mimicking acute cholecystitis. In complete volvulus, arterial supply from the cystic artery is compromised, resulting in gallbladder ischemia, necrosis, gangrene, and perforation if not recognized promptly.

Clinical manifestations

Clinical symptoms of gallbladder volvulus are variable and not specific, but they commonly mimic acute cholecystitis or acute appendicitis in elderly patients. Clinical presentation includes abdominal pain, nausea and vomiting, and a palpable mass in the right quadrant. Table 1 shows the triad of triads described by Lau et al. to recognize potential gallbladder volvulus [13]. Our presented case is unusual, as signs and symptoms of the patient suggested dyspepsia, which is not the usual presentation of gallbladder volvulus described in Table 1.

**Table 1:** Triad of triads used to recognize potential gallbladder volvulus RUQ, right upper quadrant

Appearance	Symptom	Physical examination
Elderly (usually female)	Sudden onset	Nontoxic presentation
Thin	RUQ pain	Palpable RUQ mass
Spinal deformities	Emesis	Pulse-temperature discrepancy

Diagnostic strategies

Preoperative imaging was reported to be the main contribution to the diagnosis of gallbladder volvulus. Ultrasound (US) provides morphology of markedly thickened gallbladder wall. Color Doppler can help distinguish gallbladder volvulus and gangrenous gallbladder from usual acute cholecystitis using blood flow, in which the cystic artery along the wall can be visualized in the latter condition [14]. Moreover, in gallbladder volvulus, Doppler US may demonstrate an interrupted blood flow in the cystic pedicle, which can help distinguish from gangrenous cholecystitis.

The appearance of gallbladder volvulus on CT and magnetic resonance imaging (MRI) is not specific. Many criteria were proposed to facilitate the diagnosis, which are fluid collection in the gallbladder fossa, alternative to the axis of the gallbladder from vertical to horizontal, gallbladder outside its anatomical fossa, inflammatory features of the gallbladder wall, twist along the vascular pedicle of the gallbladder with swirl appearance, or abrupt tapering of the cystic duct [4,8]. Some reports showed that the degree of gallbladder distension is greater in gallbladder volvulus than that in usual acute cholecystitis [15].

Magnetic resonance cholangiopancreatography (MRCP) is also useful for diagnosing gallbladder volvulus due to its superiority in the demonstration of the cystic duct [3,12]. However, preoperative imaging was performed in only less than half of the cases: US (50%), CT (45%), and MRI (7%). Additionally, the diagnostic yield of the condition is even less, which is reported to be 12%, 30%, and 59% of US, CT, and MRI, respectively [12].

Treatment 

Gallbladder volvulus is unlike ordinary acute cholecystitis that may respond to conservative medical treatment. Surgical intervention involving detorsion and gallbladder removal should be performed immediately to avoid gallbladder perforation and biliary peritonitis that follow [16]. Cholecystectomy can be performed by laparoscopic or open approach. The principle includes decompression, detorsion, and gallbladder removal [17]. We fully agree with the principle that the critical view of safety should be clearly identified before cholecystectomy to prevent iatrogenic bile duct injury. There was some concern about detorsion in case of gallbladder necrosis that may lead to toxin release secondary to reperfusion injury leading to systemic effect [18]. However, there was no report about reperfusion injury after detorsion in the state of gallbladder necrosis. Percutaneous gallbladder drainage should not be performed in case of gallbladder volvulus as the procedure may relieve the patient’s symptoms while continuing the ischemic process, leading to delayed treatment [3].

## Conclusions

Gallbladder volvulus is a rare but serious condition that requires a clinician’s recognition for prompt diagnosis. US is the initial imaging modality for diagnosis, and Doppler should be used in case of high suspicious index of gallbladder volvulus. Once the diagnosis of gallbladder volvulus is made, immediate surgical treatment must be performed to prevent morbidity associated with gangrenous gallbladder, gallbladder perforation, and biliary peritonitis, which would increase mortality.
